# Improving Air-Stability and Performance of Bulk Heterojunction Polymer Solar Cells Using Solvent Engineered Hole Selective Interlayer

**DOI:** 10.3390/ma11071143

**Published:** 2018-07-05

**Authors:** Binrui Xu, Gopalan Sai-Anand, Hyun-Min Jeong, Sae-Wan Kim, Ju-Seong Kim, Jin-Beom Kwon, Shin-Won Kang

**Affiliations:** 1 School of Electronics Engineering, College of IT Engineering, Kyungpook National University, 80 Daehakro, Bukgu, Daegu 41566, Korea; kezherui123@gmail.com (B.X.); hmjeong@ee.knu.ac.kr (H.-M.J.); kei95304@gmail.com (S.-W.K.); jskim5772@ee.knu.ac.kr (J.-S.K.); jinbumkwon@naver.com (J.-B.K.); 2 Global Innovative Center for Advanced Nanomaterials, Faculty of Engineering and Built Environment, University of Newcastle, Callaghan Campus, New South Wales 2298, Australia; SaiAnand.Gopalan@newcastle.edu.au

**Keywords:** solvent engineering, bulk-heterojunction, polymer solar cells, hole selective interlayer

## Abstract

In bulk heterojunction polymer solar cells (BHJ-PSCs), poly(3,4-ethylenedioxythiophene) doped with poly(styrene sulfonate) (PEDOT:PSS) is the most commonly used hole selective interlayer (HSIL). However, its acidity, hygroscopic nature, and the use of indium tin oxide (ITO) etching can degrade the overall photovoltaic performance and the air-stability of BHJ-PSCs. Solvent engineering is considered as a facile approach to overcome these issues. In this work, we engineered the HSIL using ethanol (ET) treated PEDOT:PSS to simultaneously enhance the photovoltaic performance properties and air-stability of the fabricated devices. We systematically investigated the influence of ET on the microstructural, morphological, interfacial characteristics of modified HSIL and photovoltaic characteristics of BHJ-PSCs. Compared with the BHJ-PSC with pristine PEDOT:PSS, a significant enhancement of power conversion efficiency (~17%) was witnessed for the BHJ-PSC with PEDOT:PSS-ET (*v*/*v*, 1:0.5). Consequently, the BHJ-PSC with PEDOT:PSS-ET (*v*/*v*, 1:0.5) as HSIL exhibited remarkably improved air-stability.

## 1. Introduction

**Scheme 1:**
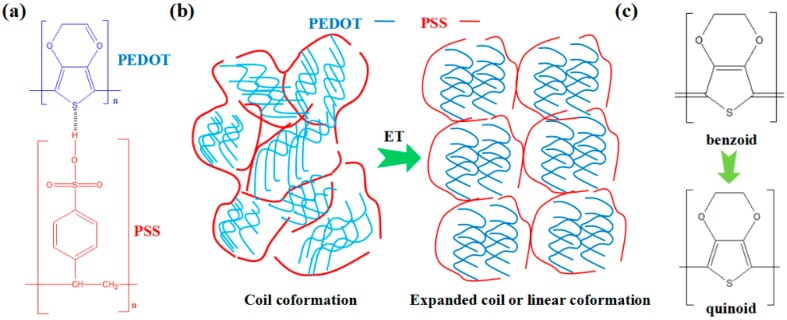
(**a**) PEDOT:PSS structure, (**b**) conformation transformation of PEDOT and PSS chains and (**c**) transformation of the PEDOT structure.

Recently, bulk-heterojunction polymer solar cells (BHJ-PSCs) have attracted widespread attention in the photovoltaics field, owing to their cost-competitiveness, mechanically flexibility, and lightweight [1,2,3]. The BHJ-PSC power conversion efficiency (PCE) has been increased over ~11% via efficient strategies on the material engineering, the addition of additives/dopants, and fabrication process [4,5]. In order to enhance photovoltaic (PV) performance of BHJ-PSCs, an interlayer was sandwiched between the electrode and the photoactive layer [6]. In existing studies, the main role of interlayer are to provide ohmic contact with the photoactive layer, transport the photoinduced carriers, select required carriers and block undesired carriers, and adjust the work function (WF) of the anode or cathode [7]. This kind of interlayer can be designated as either a hole selective interlayer (HSIL) which selects holes to pass through this layer and blocks electrons, or an electron selective interlayer (ESIL) which selects electrons and blocks holes [8]. Among the numerous conducting materials, one of the polymer materials, namely, poly(3,4-ethylene dioxythiophene):poly(styrene sulfonate) (PEDOT:PSS) becomes the most popular HSIL in BHJ-PSCs due to its favorable electrical properties and excellent optical transparency. Scheme 1a exhibits the chemical structure of PEDOT:PSS [9,10].

In general, the instability of the device hampers the industrialization of BHJ-PSCs. The instability originates from the presence of localized moisture encroachment and the interfacial passivation at the organic/cathode interface [11]. In the case of using PEDOT:PSS as HSIL, the large proportion of insulating and highly acidic PSS aggregations in PEDOT:PSS could affect the electrical conductivity of PEDOT:PSS film, corrode the indium tin oxide (ITO) electrode and damage the PV performance and air-stability of the BHJ-PSCs [12,13,14]. Therefore, numerous approaches have been used to overcome these disadvantages of PEDOT:PSS, such as PEDOT:PSS with judicial solvent modifiers [15,16]. Among the different strategies for the modification of PEDOT:PSS, incorporation of additives (e.g., dimethyl sulfoxide [17], tetramethylene sulfone [18], N,N-dimethylformamide [19], methoxyethanol [20], isopropyl alcohol [21], *N*-methyl-2-pyrrolidone [22]) effectively and drastically improves the PV performance and air-stability of BHJ-PSCs [23].

In this study, we demonstrate the ethanol (pKa = 15.9, denoted as ET) modified PEDOT:PSS (PEDOT:PSS-ET) as HSIL, and the influence on the PCE and air-stability of BHJ-PSCs. ET is a polar solvent with a hydroxyl (OH) group. These results propose that the hydroxyl group with a high electronegativity of oxygen in ET can form interaction with PEDOT chains’ dipoles or with the positive charges, and form hydrogen bonding with PSS which induces that the random coil conformation of PEDOT chains changes the ordered expanded coil/linear structure (Scheme 1b) [24]. When the above conformational change takes place, the related structural transition of PEDOT chains occurs from the benzenoid form to the quinoid form which is more conductive; this structural transformation is shown in Scheme 1c [25]. As a consequence, charge carriers can be transported faster in the expanded coil/linear conformation, enhancing the PV performance of BHJ-PSCs. Although the previous studies report ET as a useful additive in PEDOT:PSS, few studies demonstrate the influence of PEDOT:PSS-ET on the air-stability of BHJ-PSCs. The neutral nature of ET has an impact on the acidity of PEDOT:PSS, by weakening the ITO etching and improving the air-stability of BHJ-PSCs.

**Figure 1:**
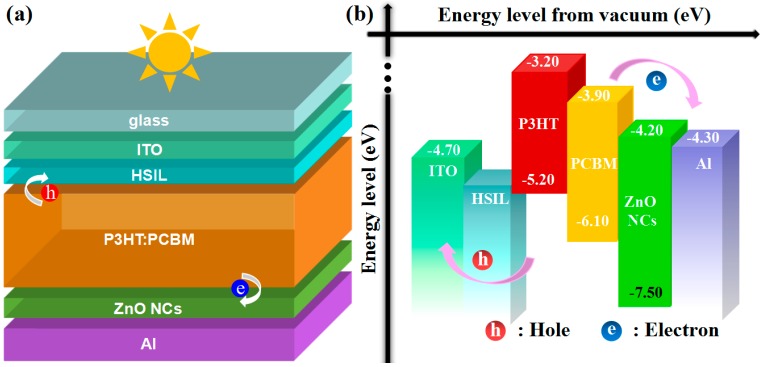
(**a**) Conventional architecture and (**b**) energy levels of compositions in BHJ-PSC.

The poly(3-hexylthiophene):[6,6]-phenyl-C61-butyric acid methyl ester (P3HT:PCBM) based BHJ-PSCs were processed according to the classical structure: glass/ITO/HSIL/P3HT:PCBM/zinc oxide nanocrystals (ZnO NCs)/aluminum (Al) (Figure 1a), and the optimized volume ratio of PEDOT:PSS-ET was found to be 1:0.5 [26,27]. Figure 1b exhibited the schematic energy band diagram of fabricated devices.

We systematically examined the influence of ET on the structure transformation, morphology modification, and surface variation of the PEDOT:PSS-ET film, as well as, the tuned WF at the interface between ITO/PEDOT:PSS-ET. By adding ET into PEDOT:PSS, an enhancement of ~17% in PCE is achieved. Subsequently, the air-stability of PEDOT:PSS-ET (*v*/*v*, 1:0.5) based BHJ-PSCs is significantly superior to the pristine device.

## 2. Materials and Methods

### 2.1. Materials and HSIL Preparation

PEDOT:PSS (Clevios P VP AI. 4083, ratio of 1:6,) was obtained from H.C. Starck, Inc., Newton, MA, USA. P3HT and PCBM were purchased from Luminescence Technology Corp., Taiwan, China, and used as received. *O*-dichlorobenzene (DCB) was obtained from Sigma-Aldrich, Seoul, Korea. ET (99.9%) was bought from Samchun Pure Chemical CO. LTD. (Pyeongtaek, Korea) and used without any purification.

The PEDOT:PSS and ET aqueous solutions were mixed with the different volume ratios of 1:0.125, 1:0.25, 1:0.375, 1:0.5, and 1:0.625. These mixtures were ultrasonicated for 1 h at room temperature.

### 2.2. Fabrication and Characterization of the BHJ-PSCs

ITO-coated glass slides were ultrasonic washing by using acetone, methanol and deionized water for 10 min, respectively [28]. Then, the ITO substrates were treated in an ultraviolet oven for 15 min. The HSILs (35 ± 5 nm) were prepared by spin-coating on the ITO substrates and thermally annealed at 150 °C for 10 min. The 20 mg of P3HT and 20 mg of PCBM were blended into 1 mL of DCB and stirred at 60 °C overnight and deposited onto the ITO/HSIL. The formed photoactive layer (~ 205 nm) was solvent annealed inside a covered glass dish for 1 h and thermally annealed at 100 °C for 10 min in a vacuum oven. Subsequently, ZnO NCs was spin-coated as ESIL on the P3HT:PCBM active layer. Finally, a top cathode of Al was thermally evaporated with an area of 9 mm^2^ at a pressure of 10^−6^ Torr. All the film fabrication processes were performed under ambient conditions (15–25% humidity in air). The PV performance parameters were measured using a 2400 series source meter (Keithley, Inc., Seoul, Korea) under 100 mW·cm^−2^ with AM 1.5 G illumination.

### 2.3. Thin Film Characterization

The transmittance spectra of HSILs coated on ITO were recorded using an ultraviolet-visible (UV-vis) spectrophotometer (Cary 5000, Agilent Technologies, Inc., Santa Clara, CA, USA). The Raman scattering spectra of HSILs was carried our using a Raman spectrometer (Model inVia, Renishaw, London, UK) with a 532-nm excitation laser line. Fourier-transform infrared (FTIR) spectra were obtained from FTIR spectroscopy (Frontier, PerkinElmer, Inc., Shanghai, China). The morphological modification of HSILs were taken using atomic force microscope (AFM, Model 5500, Agilent Technologies, Inc., Santa Clara, CA, USA). The X-ray photoelectron spectroscopy (XPS) data were evaluated by a PHI Quantera SXM scanning photoelectron spectrometer microprobe (ULVAC, Kanagawa, Japan). A contact angle analyzer (Kruss, Hamburg, Germany) was utilized to take the surface properties (water contact angle) of samples. The WFs of PEDOT:PSS and PEDOT:PSS-ET (*v*/*v*, 1:0.5) films deposited on ITO glass were determined using photoelectron yield spectroscopy (PYS) (Riken-Keiki CO., LTD, Tokyo, Japan). The acidic nature of aqueous solutions were taken by pH meter (Mettler Toledo, Shanghai, China). Field emission scanning electron microscopy (Model Hitachi SU8220, Tokyo, Japan) was used to evaluate the thickness of HSIL and photoactive layer (Figure S1).

## 3. Results

### 3.1. Photovoltaic Performance

**Figure 2:**
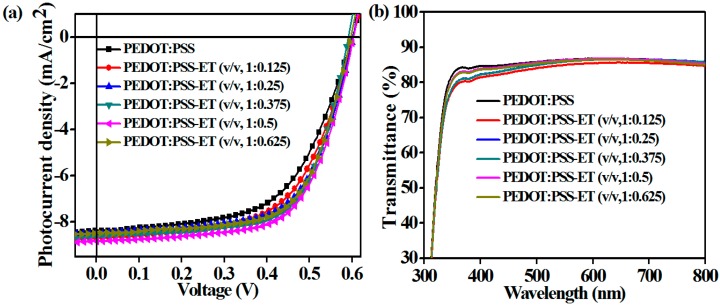
(**a**) *J-V* characteristics of the BHJ-PSCs fabricated with PEDOT:PSS and PEDOT:PSS-ET in different volume ratios, and (**b**) transmittance spectra of PEDOT:PSS and PEDOT:PSS-ET films.

The PV parameters (open-circuit voltage (*V_oc_*), short-circuit current density (*J_sc_*), series resistance (R_s_), fill factor (FF) and PCE and current density-voltage (*J-V*) curves of the fabricated BHJ-PSCs based on PEDOT:PSS or PEDOT:PSS-ET (*v*/*v*, 1:0.125, 1:0.25, 1:0.375, 1:0.5, 1:0.625) are shown in Table 1 and Figure 2a. The pristine device exhibits *V_oc_*, *J_sc_*, R_s_ and PCE values of 0.59 V, 8.376 mA/cm^2^, 241 Ω, 0.58, and 2.92%. On the other hand, the greatest PCE of 3.42% was obtained for the device with PEDOT:PSS-ET (*v*/*v*, 1:0.5) as HSIL with *V_oc_* of 0.60 V, *J_sc_* of 8.890 mA/cm^2^, FF of 0.64 and R_s_ of 155 Ω. The observable enhanced PCE is mainly due to an improvement in *J_sc_* and a decrease in R_s_. However, the devices were fabricated in ambient conditions and the degradation of the P3HT:PCBM active layer by moisture caused the high R_s_ (over 200 Ω) in this study, which affected the PV performance of BHJ-PSCs [29,30,31].

To investigate the improved performance of the BHJ-PSCs, the transmittance spectra of ITO/HSILs were recorded and are shown in Figure 2b [32,33]. The transmittance spectra of ITO/PEDOT:PSS-ET films are lower than that of the pristine ITO/PEDOT:PSS film. The transmittance spectra of ITO/PEDOT:PSS-ET (*v*/*v*, 1:0.5) and ITO/PEDOT:PSS are similar in the wavelength range of 600–800 nm; while the transmittance of ITO/PEDOT:PSS-ET (*v*/*v*, 1:0.5) film is slightly lower than the ITO/PEDOT:PSS film, from 330 nm to 600 nm [34]. Therefore, the slightly lower transmittance proves the improved PV performance of BHJ-PSCs based on PEDOT:PSS-ET films is not directly to the variant transmittance [35]. The PV performance improvement should be derive from the higher conductivity (7.89 × 10^−3^ S/cm) of PEDOT:PSS-ET thin film than that of PEDOT:PSS thin film (2.97 × 10^−3^ S/cm), resulting in the improved *J_sc_* and reduced R_s_ [36]. The enhanced electrical conductivity of PEDOT:PSS-ET film is attributed to the structural transformation of PEDOT and PSS chains [37].

### 3.2. Microstructural Properties

**Figure 3:**
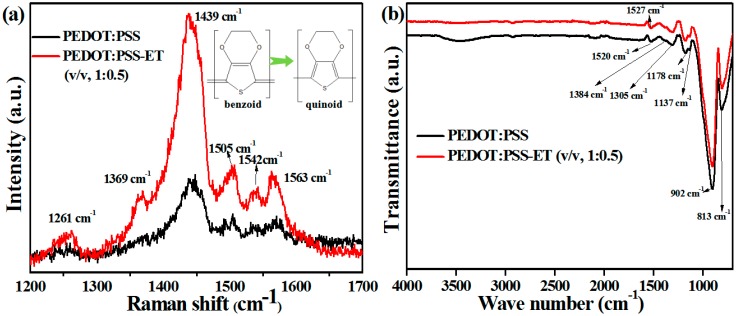
(**a**) Raman spectra of PEDOT:PSS and PEDOT:PSS-ET (*v*/*v*, 1:0.5) films on glass with, and (**b**) FTIR spectra of PEDOT:PSS and PEDOT:PSS-ET (*v*/*v*, 1:0.5) films.

In order to understand how ET impacts on the structural transformation of the PEDOT and PSS chains, Figure 3a presents the Raman data of two HSILs [38]. Both films show six predominant peaks in Raman spectra [39]. The peak at 1439, 1261, 1369, and 1542 cm^−1^ represents the C_α_=C_β_ symmetric stretching vibrations, C_α_–C_α’_ inter-ring stretching vibrations, C_β_–C_β’_ stretching vibration, and the splitting of the asymmetric vibrations, respectively. The two peaks at 1505 and 1563 cm^−1^ in the Raman spectra are corresponding with the C_α_=C_β_ asymmetric stretching vibrations which are associated with the thiophene rings in the PEDOT chains [40,41]. The intensity of the peak centered at 1439 cm^−1^ was increased, and the peak became narrower with the infusion of ET, which reveals that the benzoid-quinoid tautomerism of PEDOT chains and the conformation transformation from random coil to expand-coil/linear [42]. Due to the two PEDOT rings, with expanded coil/linear conformation, being located in almost the same plane, the π-electrons can be delocalized easily in the PEDOT rings [43]. Hence, the structural transformation of PEDOT chains presumably results in the PV performance of BHJ-PSCs.

FTIR spectrophotometer is used to evaluate the components of molecule with molecule’s characteristic absorption of infrared [44]. Figure 3b indicates the FTIR spectral characteristics of HSILs with and without ET. The peaks at 1520 and 1305 cm^−1^ are related to the C=C and C–C vibration band in the thiophene ring of PEDOT, respectively. The band at 813 and 902 cm^−1^ are assigned to the C-S bond in the EDOT ring. The stretching mode of the ethylendioxy group is centered at 1178 cm^−1^. The stretching vibration from –SO_2_ and –SO_3_ groups of PSS appeared as two weaker bands around 1137 and 1384 cm^−1^. Moreover, an important blue shift from 1520 to 1527 cm^−1^ of C=C asymmetric stretch from thiophene ring of PEDOT is noticeable, which is attributed to the delocalization of the effective π-electrons. This phenomenon corresponds to the Raman spectra, indicating the conformation transformation and structural change of PEDOT chains [42].

### 3.3. Surface Properties

**Figure 4:**
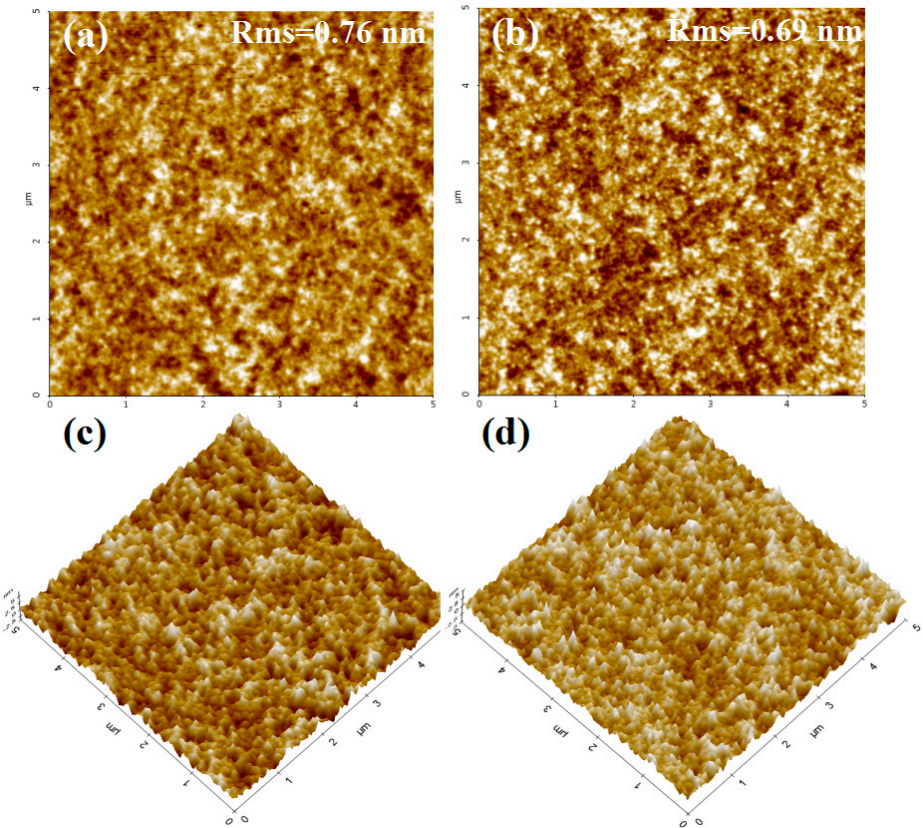
2D morphology images of (**a**) the PEDOT:PSS and (**b**) PEDOT:PSS-ET (*v*/*v*, 1:0.5) films, and 3D morphology images of (**c**) PEDOT:PSS and (**d**) PEDOT:PSS-ET (*v*/*v*, 1:0.5) films obtained with AFM at a scale of 0.5 μm × 0.5 μm.

In BHJ-PSCs, the surface morphology of HSIL has an important influence on the hole transportation. Figure 4a,b presents the surface morphological properties of PEDOT:PSS and PEDOT:PSS-ET (*v*/*v*, 1:0.5) films which were evaluated by AFM images. Three dimensional (3D) surface morphology is also exhibited in Figure 4c,d. Comparison with the root mean square roughness (RMS) of the pristine PEDOT:PSS film (0.76 nm), the AFM image of PEDOT:PSS-ET (*v*/*v*, 1:0.5) film presented a smoother, less rough surface with an RMS of 0.68 nm. The less rough surface could beneficially favor the extraction of hole collection on the ITO side [45]. Moreover, an observable phase separation between PEDOT-rich and the surrounding PSS-rich regions with the appearance of larger domains can be seen in the AFM image of the PEDOT:PSS-ET (*v*/*v*, 1:0.5), which indicates that the addition of ET can rearrange the orientation of PEDOT and PSS chains [46,47]. The obvious separation provided a larger region for continuous carrier mobility without the carriers having to hop very frequently over the insulating PSS chains, leading to the superior conductivity of PEDOT:PSS-ET (*v*/*v*, 1:0.5) film [48]. The clearer surface morphology was exhibited in the 3D AFM images [49].

**Figure 5:**
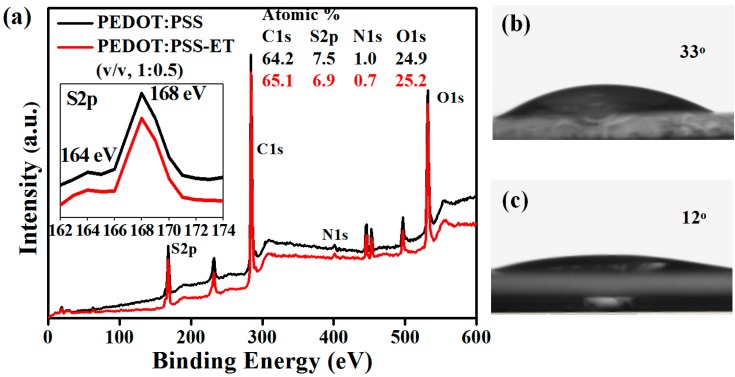
(**a**) XPS survey level C1s, S2p, N1s and O1s data of PEDOT:PSS and PEDOT:PSS-ET (*v*/*v*, 1:0.5), and water droplet contact angles of (**b**) PEDOT:PSS and (**c**) PEDOT:PSS-ET (*v*/*v*, 1:0.5) films.

To further investigate the surface chemical composition, the XPS spectra of HSILs with or without ET were recorded in Figure 5a [48,50]. The atomic weights of C1s, O1s, N1s and S2p in the XPS spectrum of pristine PEDOT:PSS film were 64.2%, O1s 24.9%, N1s 1.0% and S2p 7.5%, respectively. However, a slight decrease in the atomic weight % (6.9%) of S2p for PEDOT:PSS-ET (*v*/*v*, 1:0.5) was observed. The inserted picture presents the S2p spectra of the two films. The two binding energy peaks around 168 eV and 164 eV, and are associated with the sulfonate group from PSS and thiophene ring in PEDOT, respectively [51,52]. Due to the PEDOT to PSS ratio of 1:6, the superfluous PSS enriched on the PEDOT:PSS surface, reducing the PV performance of BHJ-PSCs. The declined S2p peaks for PEDOT:PSS-ET (*v*/*v*, 1:0.5) film revealed a decreased PSS accumulation on the surface of PEDOT:PSS-ET (*v*/*v*, 1:0.5) film, increasing the conductivity of PEDOT:PSS-ET (*v*/*v*, 1:0.5) film.

In addition, the water contact angles (Figure 5b,c) of samples have been measured to further clarify the variation of surface property in the PEDOT:PSS-ET (*v*/*v*, 1:0.5) film [53]. The water contact angle (*θ*_contact_) decreased from 33° to 12° after adding ET to the PEDOT:PSS solution [54]. This indicates that PEDOT:PSS-ET (*v*/*v*, 1:0.5) is more hydrophilic than PEDOT:PSS and PEDOT:PSS-ET (*v*/*v*, 1:0.5), indicating smoother roughness and better interface with the photoactive layer [52]. The improved interfacial adhesion is a basic factor to form a good film to fabricate the high-efficiency device.

### 3.4. Electronic Property

**Figure 6:**
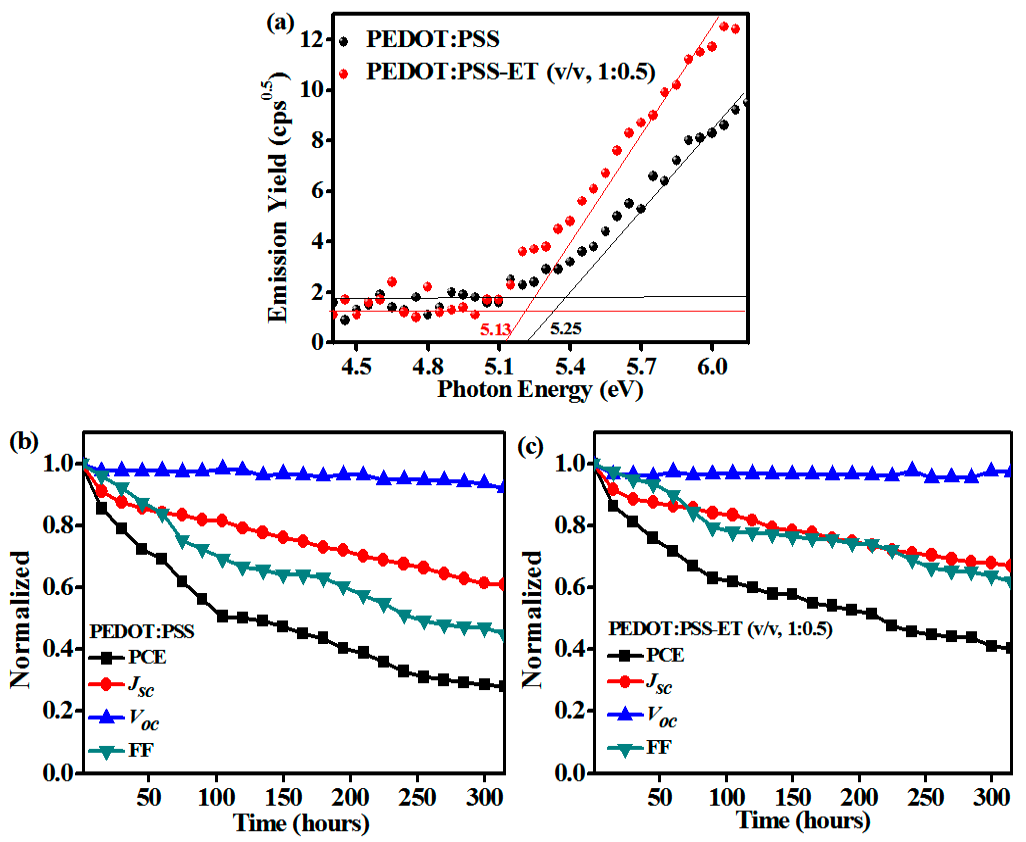
(**a**) PYS data of PEDOT:PSS and PEDOT:PSS-ET (*v*/*v*, 1:0.5) films, normalized air-stability characteristics of BHJ-PSCs with (**b**) PEDOT:PSS and (**c**) PEDOT:PSS-ET (*v*/*v*, 1:0.5) over 315 h of continuous testing.

The PYS spectra were measured to characterize the WF of ITO/HSILs as shown in Figure 6a. The WFs were extracted from the point of X-intercept as −5.25 eV (PEDOT:PSS) and −5.13 eV (PEDOT:PSS-ET (*v*/*v*, 1:0.5)), respectively [55]. The reduced PSS to PEDOT ratio at the PEDOT:PSS-ET (*v*/*v*, 1:0.5) film surface induces a variation in WF [56]. The tuned WF of PEDOT:PSS-ET (*v*/*v*, 1:0.5) in relation to the reduced PSS accumulation on the PEDOT:PSS-ET (*v*/*v*, 1:0.5) film surface [57]. The decreased content of PSS has been explained by XPS characterization. In comparison with the WF (−5.25 eV) of pristine PEDOT:PSS, the WF (−5.13 eV) is better matched to highest occupied molecular orbital (HOMO) level of P3HT (−5.20 eV) and near the WF (−4.70 eV) of ITO, which could form efficient holes transportation from active layer to the ITO electrode, leading to shortening hole extraction route and reducing the charge recombination. Consequently, holes can smoothly transport the ITO electrode, subsequently helping to improve the *J_sc_*, FF, and reducing R_s_ in the fabricated PSCs.

### 3.5. Air-Stability

The device air-stability is one of the significant parameters associated with practical utilization. To further confirm the influence of PEDOT:PSS-ET on BHJ-PSCs, the air-stability of devices with pristine PEDOT:PSS and PEDOT:PSS-ET (*v*/*v*, 1:0.5) was proposed in Figure 6b,c [58]. The PCE of pristine device decayed 72%, and the modified device decayed 60% from their initial PCE after 315 h. Furthermore, the *J_sc_* of the pristine device reduced by 39% and the FF dropped by 55% after 315 h. However, ~33% of the *J_sc_* and ~39% of FF were dropped in the device with PEDOT:PSS-ET (*v*/*v*, 1:0.5). Simultaneously, the *V_oc_* of both two devices showed a similar linear trend. The decreased PCE in both BHJ-PSCs is related to the obvious reduced *J_sc_* and FF. In conclusion, the device with PEDOT:PSS-ET (*v*/*v*, 1:0.5) exhibited significantly improved air-stability and longer lifetime than the pristine device. The corrosion of the ITO electrode and the active layer by acidic PEDOT:PSS is a major cause of poor air-stability of the device [59]. Therefore, we envisage that the reason for better air-stability in the completed BHJ-PSCs with PEDOT:PSS-ET (*v*/*v*, 1:0.5) is ethanol, which could neutralize or reduce the acidity of the PSS. The pH values of PEDOT:PSS solutions with various volume ratios of ET were measured by the pH meter, as shown in Table 2. With the increased volume ratio of ET, the pH value of PEDOT:PSS was gradually increased. Due to the optimized pH value, the etching of the ITO electrode and the blend active system caused by PEDOT:PSS, can be controlled. Consequently, the air-stability of the device with PEDOT:PSS-ET (*v*/*v*, 1:0.5) was significantly improved.

Herein, with the enhanced properties of PEDOT:PSS-ET (*v*/*v*,1:0.5) film, the PCE of BHJ-PSCs were significantly increased from 2.92% to 3.42%; as well as the improved PV parameters, such as the improved *J_sc_* (from 8.376 to 8.890 mA/cm^2^), the enhanced FF ( from 0.58 to 0.64) and the reduced R_s_ (241 to 155 Ω). The improved PCE is mainly attributed to the improved *J_sc_*, induced by the conformation transform of PEDOT and PSS chains. Moreover, due to the pH value of the ET (nearly to 7), the device with PEDOT:PSS-ET (*v*/*v*, 1:0.5) exhibited excellent air-stability after aging 315 h, as compared with the device with pristine PEDOT:PSS.

## 4. Conclusions

In this work, BHJ-PSCs based on solvent engineered HSIL were fabricated and appropriately characterized. By introducing an inexpensive solvent (ethanol, ET) as a solvent additive in the PEDOT:PSS, the conformation of PEDOT:PSS can be transformed from a random coil form to a more-ordered expanded coil/linear form; the resulting PEDOT:PSS-ET exhibited better conductivity, more favorable surface morphology, reduced PSS content and suitable work function with the donor polymer. With these advantageous properties, the fabricated BHJ-PSCs employing PEDOT:PSS-ET (*v*/*v*, 1:0.5) achieved an increment of ~17% in the PCE. Moreover, the utilized ET can control the acidity of PEDOT:PSS to avoid the corrosion and degradation of the ITO electrode and the active layer, significantly improving the air-stability and performance of the device.

## Figures and Tables

**Table 1:** PV parameters of the BHJ-PSCs fabricated with PEDOT:PSS and PEDOT:PSS-ET in different volume ratios. The average parameters were obtained from eight independent devices. PCE^a^ and PCE^b^ are the best and average PCE values, respectively.

HSIL	*V_oc_* (V)	*J_sc_* (mA/cm^2^)	R_s_ (Ω)	FF	PCE^a^ (%)	PCE^b^ (%)
pristine	0.59 ± 0.006	8.376 ± 0.07	241 ± 9.3	0.58 ± 0.016	2.92	2.86 ± 0.094
1:0.125	0.59 ± 0.004	8.421 ± 0.04	195 ± 8.5	0.59 ± 0.006	3.10	3.08 ± 0.012
1:0.25	0.60 ± 0.004	8.565 ± 0.03	170 ± 3.7	0.63 ± 0.005	3.22	3.21 ± 0.015
1:0.375	0.60 ± 0.004	8.652 ± 0.06	162 ± 4.6	0.63 ± 0.007	3.33	3.30 ± 0.016
1:0.5	0.60 ± 0.005	8.890 ± 0.05	155 ± 5.8	0.64 ± 0.004	3.42	3.36 ± 0.041
1:0.625	0.59 ± 0.005	8.572 ± 0.08	159 ± 6.4	0.63 ± 0.005	3.27	3.22 ± 0.035

**Table 2:** pH values of PEDOT:PSS and PEDOT:PSS-ET aqueous solutions.

PEDOT:PSS-ET (*v*/*v*)	pH Value
Pristine	1.65
1:0.125	1.77
1:0.25	1.96
1:0.375	1.99
1:0.5	2.04
1:0.625	2.14
ET	6.87

## Supplementary Materials

The following are available online at http://www.mdpi.com/1996-1944/11/7/1143/s1, Figure S1: FE-SEM cross-section image of (**a**) the completed device, (**b**) ITO/PEDOT:PSS and (**c**) ITO/PEDOT:PSS-ET (*v*/*v*, 1:0.5).
