# Supplementary material for: Improving Air-Stability and Performance of Bulk Heterojunction Polymer Solar Cells Using Solvent Engineered Hole Selective Interlayer
Source: Materials (Basel). 2018 Jul 5;11(7):1143. doi: 10.3390/ma11071143 (PMC6073352; doi:10.3390/ma11071143)
Supplement: Supplementary file 1 [file materials-11-01143-s001.pdf]

Improving Air-stability and Performance of Bulk Heterojunction Polymer Solar Cells Using Solvent Engineered Hole Selective Interlayer

Binrui Xu [1], Gopalan Sai-Anand [1,2], **Hyun-Min Jeong** [1], Sae-Wan Kim [1], Jin-Beom Kwon [1] and Shin-Won Kang [1,*]

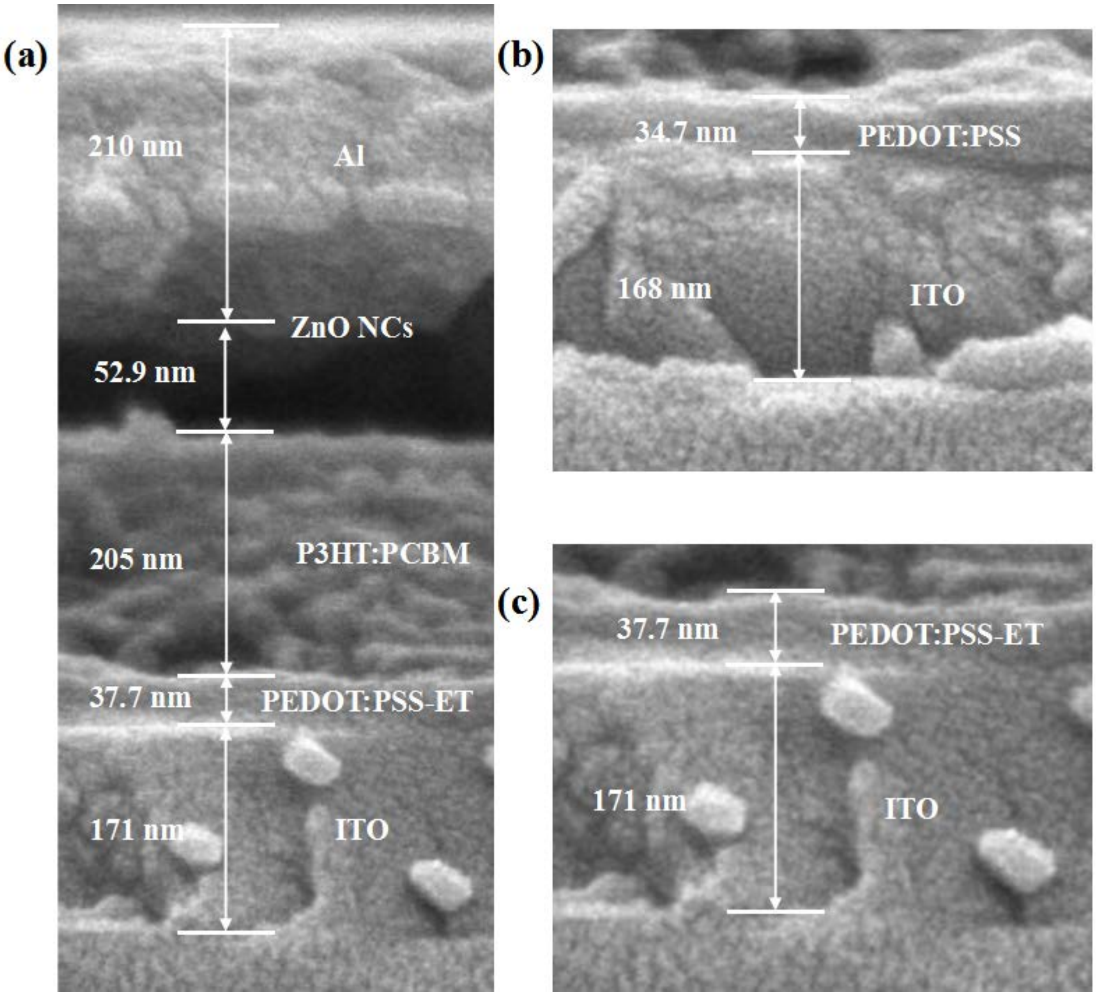

Figure S1. FE-SEM cross-section image of (a) the completed device, (b) ITO/PEDOT:PSS and (c) ITO/PEDOT:PSS-ET (v/v, 1:0.5).

Figure S1 exhibits the Field Emission Scanning Electron Microscopy (FE-SEM) cross-section image of fabricated device with the structure of glass/ITO/poly(3,4-ethylenedioxythiophene):poly(styrene sulfonate)-ethanol (PEDOT:PSS-ET)/ poly(3-hexylthiophene): [6,6]-phenyl-C61-butyric acid methyl ester (P3HT:PCBM) /zinc oxide nanocrystals (ZnO NCs)/Al. It can be seen from Figure S1 (b-c), These two films shows similar thickness, the thickness of PEDOT:PSS is near 34.7 nm and the thickness of PEDOT:PSS-ET (v/v, 1:0.5) is 37.7 nm.
